# Dabrafenib in an elderly patient with metastatic melanoma and *BRAF* V600R mutation: a case report

**DOI:** 10.1186/s13256-016-0953-0

**Published:** 2016-06-02

**Authors:** David Casadevall, Joana Vidal, Fernando Gallardo, Flavio Zuccarino, Montserrat Arumí-Uría, Alba Dalmases, Beatriz Bellosillo, Clara Montagut

**Affiliations:** 1 Medical Oncology Department, Hospital del Mar, Passeig Marítim 25-29, 08003 Barcelona, Spain; 2 Dermatology Department, Hospital del Mar, Barcelona, Spain; 3 Radiology Department, Hospital del Mar, Barcelona, Spain; 4 Pathology Department, Hospital del Mar, Barcelona, Spain; 5 Cancer Research Program, IMIM (Hospital del Mar Medical Research Institute), Barcelona, Spain

**Keywords:** Melanoma, BRAF, V600R, Dabrafenib, ctDNA

## Abstract

**Background:**

Approximately 50 % of malignant melanomas harbor activating point mutations in the *BRAF* gene. Typically, these mutations result in the substitution of the amino acid valine at codon 600 of the gene, and 90–95 % of mutations are either *BRAF* ^V600E^ or *BRAF* ^V600K^. Specific BRAF inhibitors such as dabrafenib and vemurafenib are the mainstays of treatment in patients with metastatic *BRAF*-mutant malignant melanomas. The third most common *BRAF* mutation is V600R, which also leads to increased BRAF signaling. Although evidence exists about the activity of dabrafenib and vemurafenib in patients with the *BRAF* ^V600R^ mutation, these patients have been systematically excluded from recent trials with targeted therapies.

**Case presentation:**

Here, we report the positive results in terms of survival and quality of life obtained with dabrafenib in an 80-year-old Caucasian male patient with a Charlson Comorbidity Index of 8 diagnosed with metastatic malignant melanoma harboring the *BRAF* ^V600R^ mutation. Our patient was treated with dabrafenib for 7 months with minimal toxicity. We also report exploratory analyses of circulating tumor DNA during targeted treatment. Interestingly, the mutation was not detected after starting treatment and became detectable before radiological disease progression.

**Conclusions:**

Our report suggests that (1) a relevant benefit can be obtained with a BRAF inhibitor in real-world patients with a malignant melanoma harboring a *BRAF* ^V600R^ mutation, and that (2) circulating tumor DNA detection might be of help in assessing tumor burden in everyday clinical practice. The results reported here should encourage the inclusion of patients with *BRAF* ^V600R^-mutated malignant melanomas in future prospective clinical trials with BRAF inhibitors.

## Background

The *BRAF* gene is localized at 7q34 and encodes a family of protein kinases involved in cell signal transduction through the mitogen-activated protein kinase MAPK (Ras/Raf/MEK/ERK) pathway, which mainly affects cell differentiation and proliferation [1–4]. Activation of the MAPK pathway, generally through point mutations in one or more of its components, has been associated with several diseases, such as cardiofaciocutaneous syndrome, Erdheim-Chester disease, Langerhans cell histiocytosis, and Noonan syndrome [2–4]. *BRAF* mutations have also been associated with malignancies, such as melanoma, thyroid carcinoma, and colorectal carcinoma [5, 6].

Identification of BRAF as an actionable target in advanced malignant melanoma (MM) has led to a dramatic change in the treatment scope of this disease [7]. Approximately 50 % of MMs harbor mutations in amino acid 600 of the *BRAF* gene [5]. These are usually amino acid substitutions that result in a protein conformation change that leads to constitutionally elevated kinase activity [6]. Over 90 % of activating mutations are either valine-glutamate (*BRAF* ^V600E^) or valine-lysine (*BRAF* ^V600K^) substitutions, which account for 75 and 15 %, respectively [8]. Dabrafenib and vemurafenib were the first BRAF inhibitors to show a clear benefit over chemotherapy treatment in patients with *BRAF*-mutated metastatic MM [9, 10].

Although initially designed to target the *BRAF* ^V600E^ mutation, early studies of dabrafenib and vemurafenib also included *BRAF* ^V600K^-mutated melanoma cases, and showed a clinical benefit in this subset of patients [7, 11, 12]. The third most frequent *BRAF* mutation is V600R, accounting for 1–5 % depending on the series and sequencing technique [8, 13, 14]. Patients with this rare mutation were excluded from pivotal trials with BRAF inhibitors and have also been excluded from the more recent BRAF/MEK dual inhibition trials [15, 16]. However, activity of BRAF inhibitors in this context has been reported in BRAF^V600R^-mutated melanoma cell lines [17] and in a small Australian patient series [18].

Here, we present a case of durable clinical benefit with the use of dabrafenib in an 80-year-old patient with moderate comorbidity diagnosed with metastatic MM harboring the *BRAF* ^V600R^ mutation. Additionally, due to the emergence of circulating tumor (ct) DNA as a promising biomarker for assessing tumor burden and treatment response [19], we collected serial plasma samples and report exploratory analyses of ctDNA during our patient’s treatment.

## Case presentation

Our patient was an 80-year-old Caucasian man with a history of arterial hypertension, dyslipidemia, hyperuricemia, and hypothyroidism, for which he was being medically treated. He did not drink alcohol and was a former smoker of cigarettes. Our patient had no familiar history of cancer. He had undergone a right nephrectomy in 2002 due to a spontaneous retroperitoneal hematoma and consequently had chronic renal insufficiency (usual creatinine levels of 1.6–2.0 mg/dL and a glomerular filtration rate of 40 mL/min). He had no history of cognitive impairment or dementia and retained full autonomy in his daily activities and personal care needs. In June 2010 he had undergone surgical resection of a 26 mm-diameter pigmented lesion on his right pre-auricular region. The pathology report disclosed an ulcerated MM with a Breslow index of 0.7 mm and a Clark level of III (pT1b, Stage IB) [20].

### Clinical findings

**Fig. 1:**
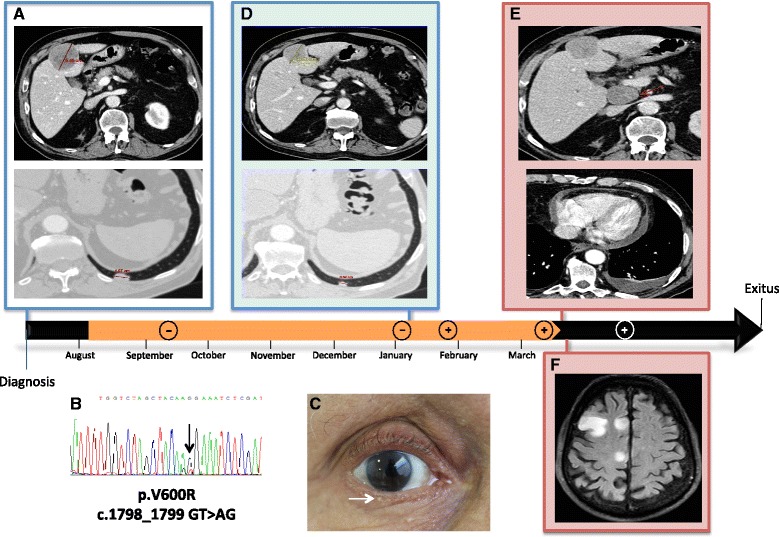
Timeline. The *black arrow* represents time from diagnosis of metastatic disease until patient’s death. The *orange arrow* represents the duration of treatment with dabrafenib (7.1 months). Along the arrows, the − and + symbols represent the time points at which the plasma *BRAF* ^V600R^ mutation was negative and positive, respectively. **a** Initial CT scans (21 June 2013) showing hepatic (*top*) and pulmonary (*bottom*) lesions. **b** Representation of V600R mutation detected by Sanger sequencing. The black arrow points to the nucleotide changes. **c** Appearance of milium cysts (white arrow) secondary to dabrafenib treatment. **d** CT scans (8 January 2014) showing the radiological response is maintained after 5 months of treatment. The pictures correspond to the same lesions shown in a. **e** CT scans (17 March 2014) showing disease progression after 7.1 months of dabrafenib treatment. *Top* picture shows a new right adrenal mass and the *bottom* picture reveals the appearance of pericardial and pleural effusions. **f** Brain MRI showing multiple brain metastases (1 April 2014)

When first evaluated by our Oncology Department, our patient presented with moderate asthenia that limited his daily activity, without other relevant clinical symptoms. A physical examination did not detect any relevant findings (Eastern Cooperative Oncology Group [ECOG] performance status of 1). A full-body CT scan revealed the presence of pulmonary and hepatic nodules (Fig. 1a). No brain metastases were detected. A complete blood work-up produced the following results (normal range values in parenthesis): glucose 87 mg/dL (75–115), urea 70 mg/dL (10–50), serum creatinine 1.76 mg/dL (0.6–1.4), glomerular filtration rate 40 mL/min/1.73m^2^ (>60), urate 7.4 mg/dL (3.4–7.0), sodium 138 mmol/L (135–146), potassium 4.7 mmol/L (3.5–5.1), total bilirubin 0.43 mg/dL (0.2–1.2), aspartate aminotransferase (AST) 59 UI/L (10–38), alanine aminotransferase (ALT) 52 UI/L (7–41), gamma-glutamyltransferase (GGT) 363 UI/L (8–61), alkaline phosphatase 204 UI/L (40–129), serum calcium 9 mg/dL (8.5–10.5), serum albumin 3.9 g/dL (3.8–5.1), hemoglobin 12.1 g/dL (13–17), total leukocyte count 10.1 × 10^3^/μL (4–11), neutrophil count 6.5 × 10^3^/μL (2.5–8.2), lymphocyte count 1.6 × 10^3^/μL (1.5–5), and total platelet count 290 × 10^3^/μL (150–450). It is important to highlight that his LDH levels were elevated (820 UI/L) [21].

### Diagnostic assessment and therapeutic intervention

Our patient underwent a core-needle biopsy of the largest hepatic lesion in segment IVb without any complications. Pathology results were positive for MM and therefore the biopsy specimen was studied for mutations in *BRAF*, *KIT*, and *NRAS* genes using the cobas 4800 Test (cobas® 4800 BRAF V600 Mutation Test; Roche Molecular Diagnostics, Inc., Pleasanton, CA, USA) and Sanger sequencing for the three genes. The V600R mutation was detected in the *BRAF* gene (Fig. 1b); no mutations were detected in the *KIT* or *NRAS* genes. In August 2013, our patient was started on dabrafenib treatment in a compassionate-use program at the initial standard dose of 150 mg orally every 12 h.

### Follow-up and outcome

Our patient’s asthenia showed a rapid recovery, with rapid improvement in his ECOG score from 1 to 0 in the first month of treatment. In subsequent evaluations, only minimal cutaneous toxicity appeared (beard alopecia and the appearance of several milium cysts, see Fig. 1c). Several warty lesions appeared, none of them compatible with keratoacanthoma or cutaneous squamous-cell carcinoma.

His first radiological evaluation by CT, 2.5 months after starting treatment, showed a partial reduction of both the hepatic and pulmonary lesions, which was considered to indicate stable disease by Response Evaluation Criteria In Solid Tumors (RECIST) 1.1 criteria [22]. This radiologic response was maintained in a second CT evaluation after 5 months of therapy (Fig. 1d).

Seven months after the start of treatment, our patient presented with pneumonia and was admitted to our Oncology ward. During his stay at our hospital, a CT scan showed hepatic and retroperitoneal progression, as well as the appearance of pericardial and pleural effusions (Fig. 1e). Additionally, MRI detected multiple bilateral brain metastases (Fig. 1f). Treatment with dabrafenib was stopped. His brain lesions were considered unresectable, and our patient received whole brain radiotherapy. After this, he maintained an ECOG performance status of 2 and was thus deemed non-eligible for further treatments. He was referred to our palliative care unit and received regular in-home visits. Our patient died in September 2014.

### Exploratory ctDNA analysis

As part of a research project at our center, plasma samples were periodically extracted during therapy. For this patient, five plasma samples were available for *BRAF* mutation analysis. Plasma was obtained from 8 mL of peripheral blood collected in tubes with EDTA as the anticoagulant. The plasma was separated within 5 h by centrifugation at room temperature for 15 min at 3200 rpm, then aliquoted and stored at −80 °C. ctDNA was extracted from the plasma using the QIAamp Circulating Nucleic Acid Kit (Qiagen, Hilden, Germany) according to the manufacturer’s instructions. The *BRAF* ^V600R^ mutation was targeted in a real-time 7500 fast PCR (Life Technologies, Foster City, CA, USA) using a competitive allele-specific TaqMan (cast-PCR) assay specifically designed for this mutation (Life Technologies).

In the samples extracted on 13 September 2013 and 7 January 2014, the mutation was undetectable. In the samples extracted on 30 January and 13 March 2014 (the latter coinciding with the CT scan showing disease progression), the tumor’s known *BRAF* ^V600R^ mutation was detected. The mutation was further detected in the samples extracted in April and May 2014.

## Discussion

The *BRAF* ^V600R^ mutation occurs in up to 5 % of MM cases and it results in the substitution of valine by arginine at amino acid 600 of the *BRAF* gene. Similar to *BRAF* ^V600E^ and *BRAF* ^V600K^, *BRAF* ^V600R^ causes an increase in *BRAF* protein-kinase activity [13, 23]. Evidence regarding the clinical activity of BRAF inhibitors in patients with tumors harboring the *BRAF* ^V600R^ mutation is scarce and comes from case reports and series of cases. Klein *et al*. [18] have reported the largest series of patients with *BRAF* ^V600R^-mutant tumors treated with BRAF inhibitors. Five out of nine patients in this series showed a partial response and one patient was maintained for 10.4 months on dabrafenib treatment before showing disease progression. In the same year, van den Brom *et al*. [24] reported clinical activity of vemurafenib in a patient with a *BRAF* ^V600R^-mutated MM with a solitary brain metastasis. They observed a significant improvement in their patient’s neurological symptoms and demonstrated a partial response of the brain lesion by RECIST criteria.

Our patient was evaluated as an 80-year-old man with an excellent quality of life, with an ECOG performance status of 1 with moderate comorbidity (age-adjusted Charlson Index of 8) and a poor prognosis according to total LDH levels [21]. At our institution, *BRAF* mutations are initially assessed through the cobas 4800 Test and all cases are further confirmed by Sanger sequencing. At the time of our patient’s diagnosis, approval of dabrafenib or vemurafenib in Spain by the national regulatory agencies (AEMPS) was still pending. In our public health system, the only authorized first-line treatments were chemotherapeutic agents such as dacarbazine or fotemustine. Clinical benefits with chemotherapy are only seen in a limited number of patients. In the most recent trials in patients with *BRAF*-mutated MM being treated with dacarbazine in the control arm, response rates were around 5–10 % and progression-free survival (PFS) times were less than 2 months [7, 10, 25]. After discussing the benefits and risks of chemotherapy versus a targeted agent with the patient and his family, a decision was made for a targeted therapy. We were able to obtain dabrafenib in a compassionate-use protocol from GlaxoSmithKline. In the pivotal trial of dabrafenib [10], elderly patients were also included, and the maximum age was 93 in the experimental arm. Also, dabrafenib is mainly eliminated in the feces and thus needed no dose adjustment considering our patient’s renal function.

As reported above, our patient’s tolerance to the treatment was excellent, with minimal cutaneous toxicity. He stayed on the treatment for 7.1 months, which is above the median PFS observed in the BREAK-3 trial [10], and he lived for 15 months.

Non-*BRAF* ^V600E^ mutations have been associated with increasing age, and clinical differences have been reported regarding the primary site of the tumor between *BRAF* ^V600E^- and *BRAF* ^V600K^-mutated MMs [26]. Interestingly, the nine *BRAF* ^V600R^-mutated MMs reported by Klein and collaborators [18] share some clinical features with our patient. Eight of their nine patients were men and four of them were older than 70 years. Primary lesions were in the scalp in four patients and were ulcerated in six of them. However, the numbers are too small to draw conclusions.

Finally, we performed an exploratory analysis of ctDNA as a biomarker during the targeted treatment. Although technical and economical limitations preclude us from using this method efficiently in our daily clinical practice, it shows great promise as a means for early diagnosis and tumor-burden monitoring [19, 27]. All our analyses were performed retrospectively within an investigational trial and were not used for treatment decisions. In our patient, the mutation was undetectable during dabrafenib treatment and response, becoming detectable before radiologically detected disease progression. Although it is tempting, no robust conclusions can be drawn from these observations, since we had no available plasma at the time of diagnosis and we did not explore other mutations apart from the *BRAF* ^V600R^. However, this finding is coherent with the knowledge that, in MM with acquired resistance to BRAF inhibitors, the original *BRAF* mutation persists [28].

## Conclusions

This case report highlights (1) the benefits that can be obtained with a BRAF inhibitor in real-world patients with MM harboring the *BRAF* ^V600R^ mutation, and (2) the potential application of emerging new techniques such as ctDNA detection in our everyday clinical practice. In our opinion, the possibility of on-site molecular testing and the availability of targeted treatment greatly impacted our patient’s outcome and quality of life.

Finally, patients with an MM harboring a *BRAF* ^V600R^ mutation should not be excluded from randomized clinical trials (RCTs). This is of greatest importance in countries with a public health system, because local regulatory agencies strictly rely on RCT data to approve new treatments for the different subsets of patients.

## Abbreviations

ALT, alanine aminotransferase; ASP, aspartate aminotransferase; CT, computed tomography; ctDNA, circulating tumor DNA; ECOG, Eastern Cooperative Oncology Group; ERK, extracellular signal-regulated kinases; GGT, gamma-glutamyltransferase; LDH, lactate dehydrogenase; MM, malignant melanoma; MRI, magnetic resonance imaging; PFS, progression-free survival; RAF, raf proto-oncogene;RAS, rat sarcoma oncogene; RCT, randomized controlled trial; MEK, mitogen-activated protein kinases / extracellular signal-regulated kinases
